# Supplementary material for: Synergism of a Novel 1,2,4-oxadiazole-containing Derivative with Oxacillin against Methicillin-Resistant Staphylococcus aureus
Source: Antibiotics (Basel). 2021 Oct 16;10(10):1258. doi: 10.3390/antibiotics10101258 (PMC8532612; doi:10.3390/antibiotics10101258)

Supplementary data

Synergistic activity of a synthetic 1,2,4-oxadiazole-containing derivative and oxacillin against methicillin-resistant *Staphylococcus aureus*

Elisabetta Buommino [1, †], Simona De Marino [1, †], Martina Sciarretta [1], Marialuisa Piccolo [1], Carmen Festa [1,*] and Maria Valeria D'Auria [1]

Table of contents

1. ^{1}H spectrum, mass spectrum and HPLC chromatogram of compound **3** 2
2. ^{1}H and ^{13}C NMR spectra of compound **4** 3
3. ^{1}H and ^{13}C NMR spectra of compound **5** 4
4. ^{1}H and ^{13}C NMR spectra of compound **6** 5
5. ^{1}H and ^{13}C NMR spectra of compound **7** 6
6. ^{1}H and ^{13}C NMR spectra of compound **8** 7
7. ^{1}H and ^{13}C NMR spectra of compound **9** 8
8. ^{1}H and ^{13}C NMR spectra of compound **10** 9
9. ^{1}H and ^{13}C NMR spectra of compound **11** 10
10. ^{1}H and ^{13}C NMR spectra of compound **12** 11
11. Mass spectrum and HPLC chromatogram of compound **12** 12
12. ^{1}H and ^{13}C NMR spectra of compound **13** 13
13. ^{1}H and ^{13}C NMR spectra of compound **14** 14
14. ^{1}H and ^{13}C NMR spectra of compound **15** 15
15. ^{1}H and ^{13}C NMR spectra of compound **16** 16
16. ^{1}H and ^{13}C NMR spectra of compound **17** 17

^{1}H NMR of compound **3** (400 MHz, CD_3OD)

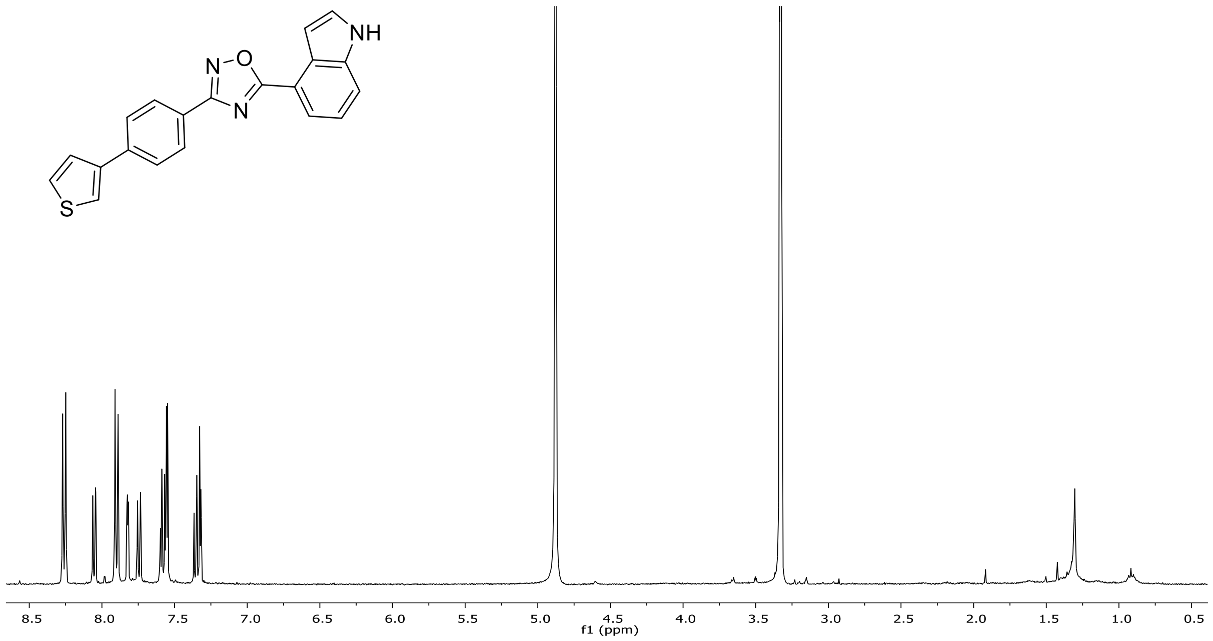

ESIMS spectrum of compound **3** (in positive mode)

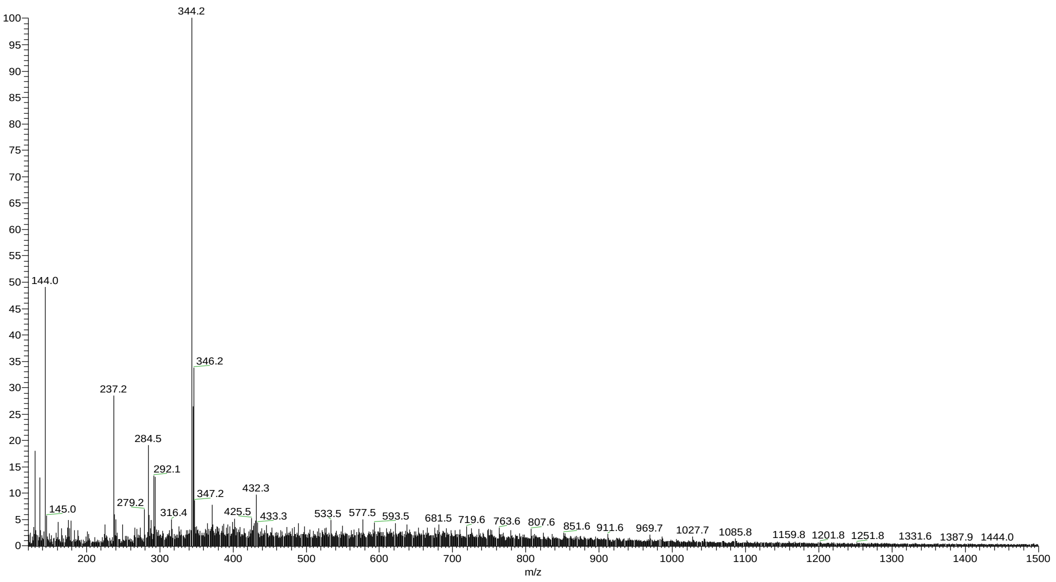

HPLC chromatogram of compound **3**

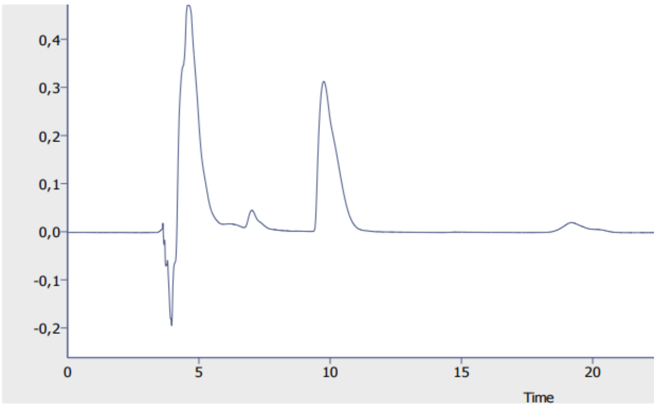

^{1}H NMR of compound **4** (700 MHz, CD_3OD)

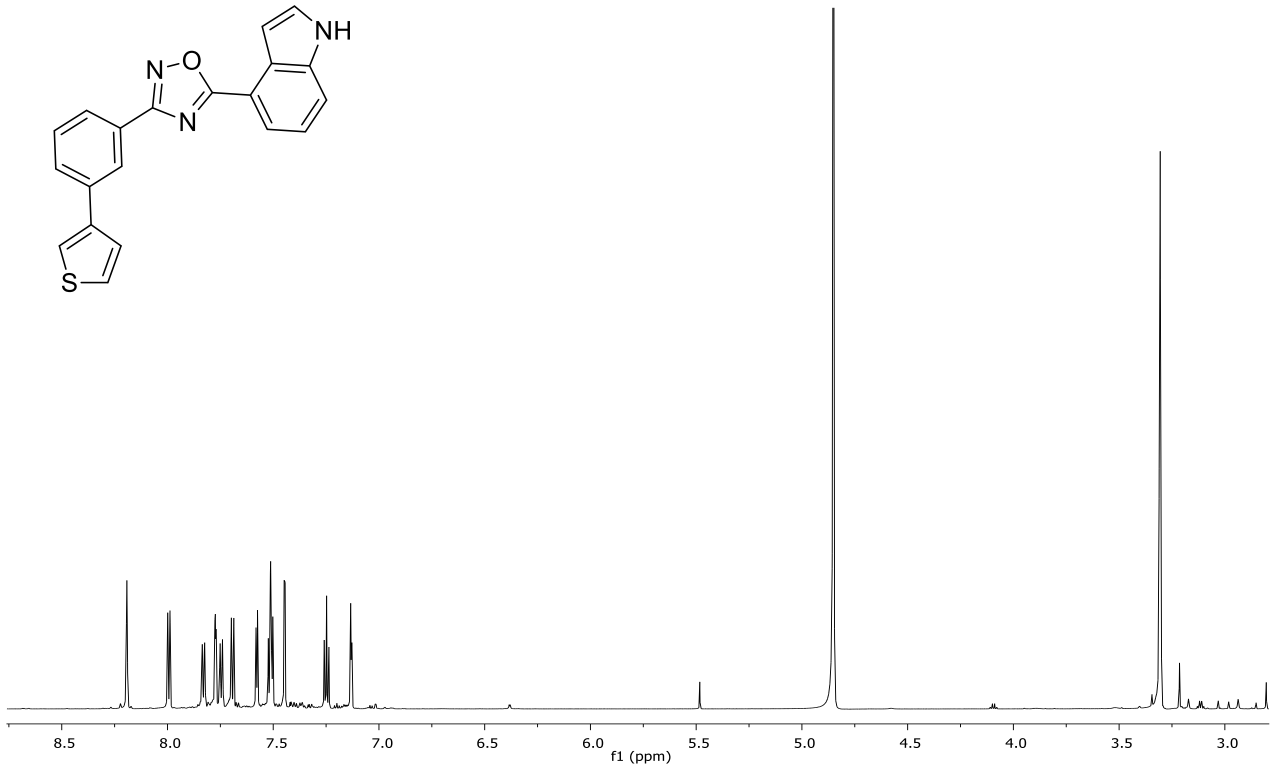

^{13}C NMR of compound **4** (175 MHz, CD_3OD)

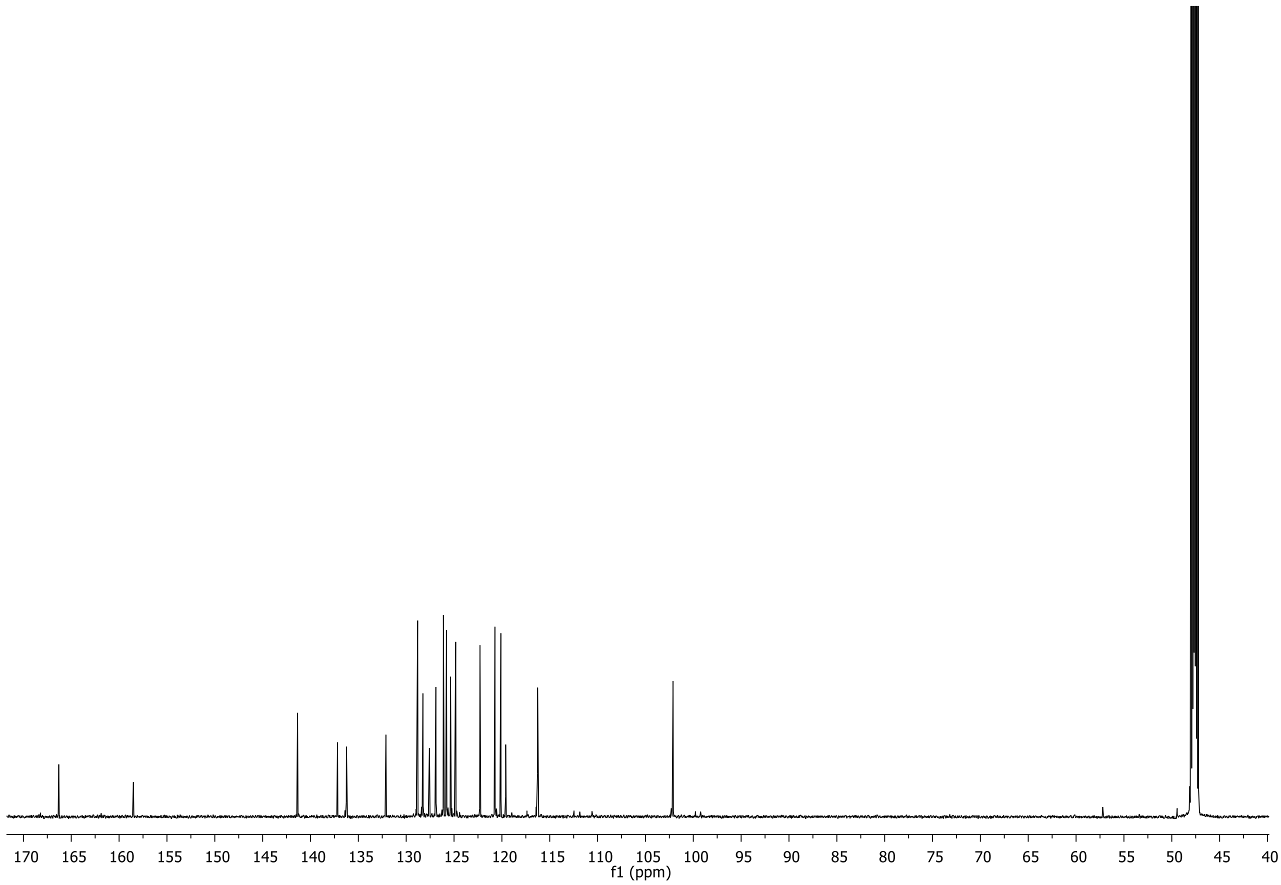

^{1}H NMR of compound **5** (700 MHz, CD_3OD)

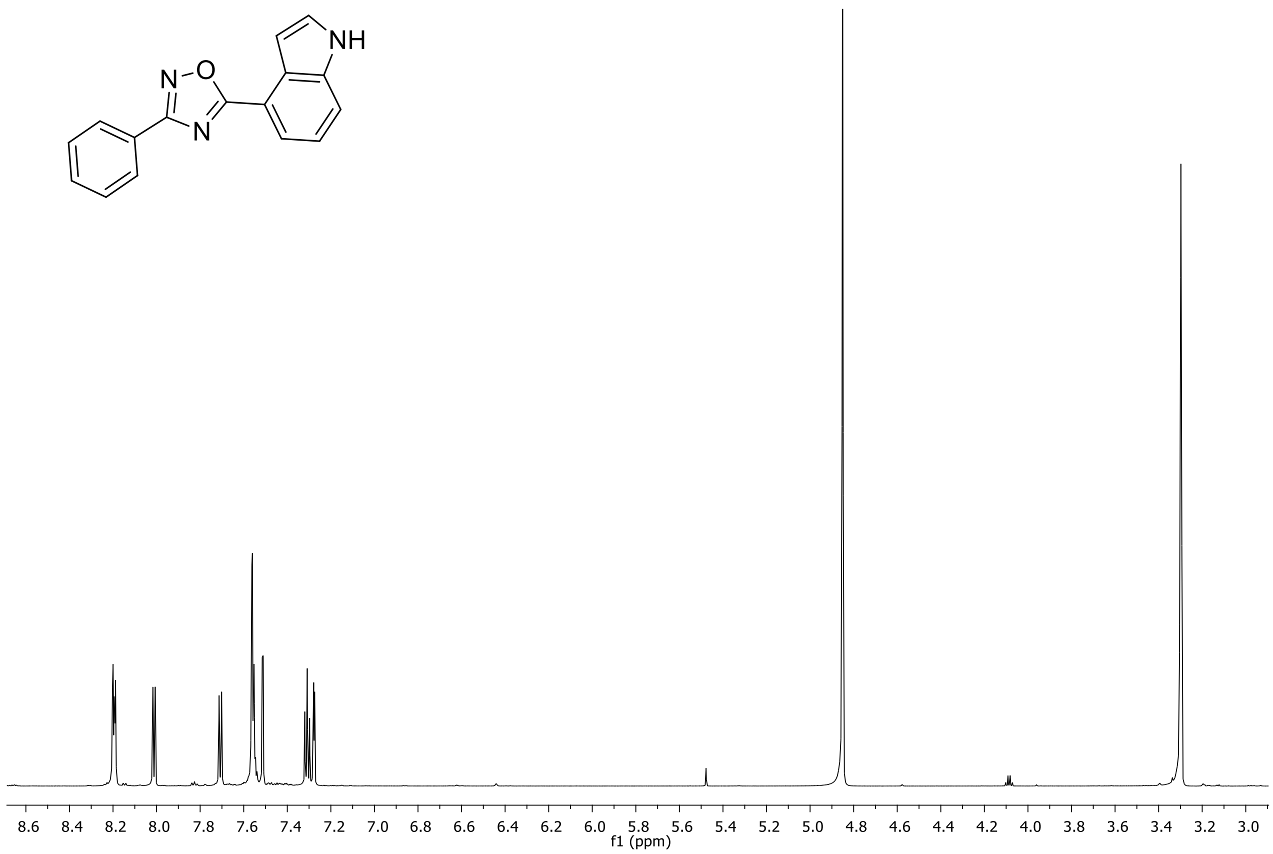

^{13}C NMR of compound **5** (175 MHz, CD_3OD)

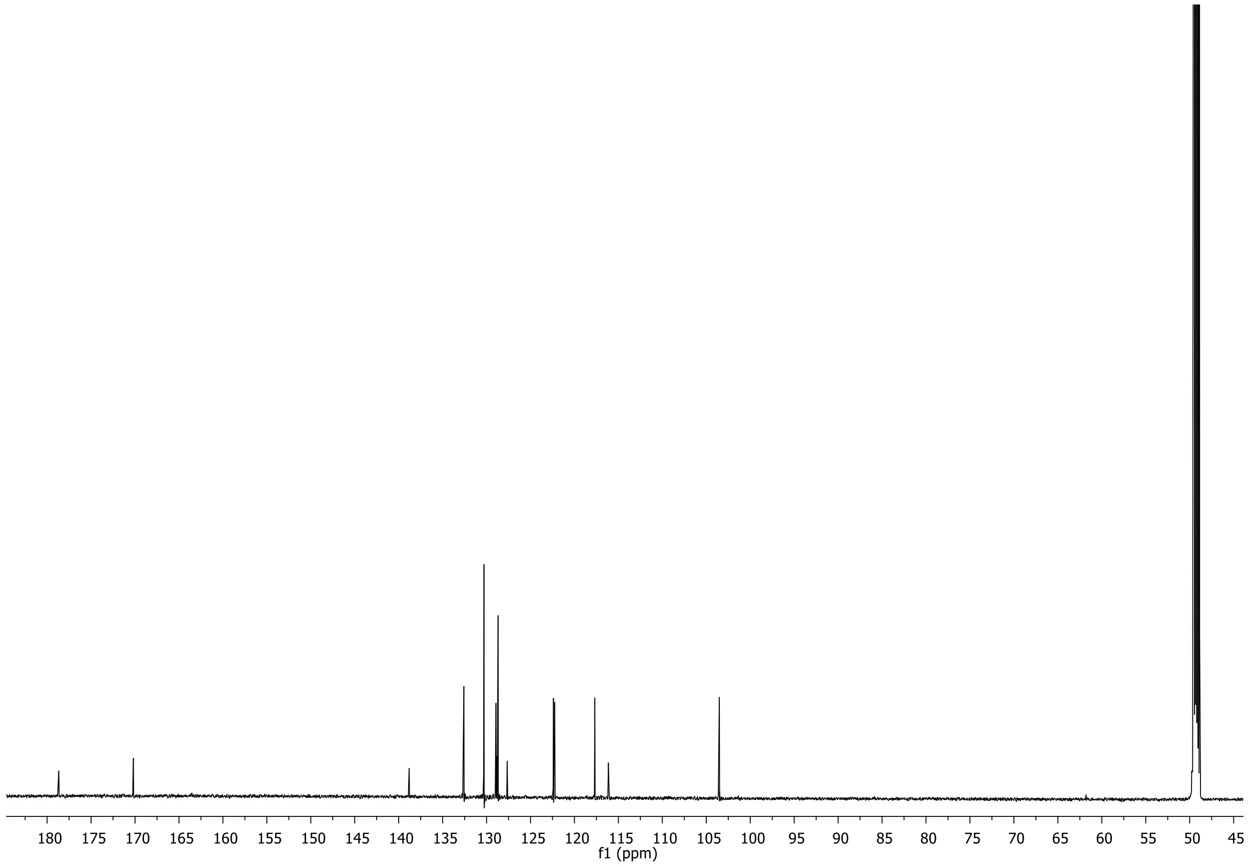

^{1}H NMR of compound **6** (700 MHz, CD_3OD)

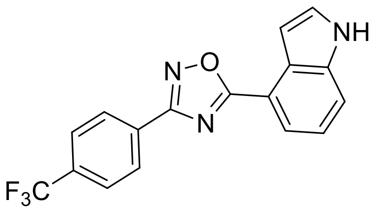

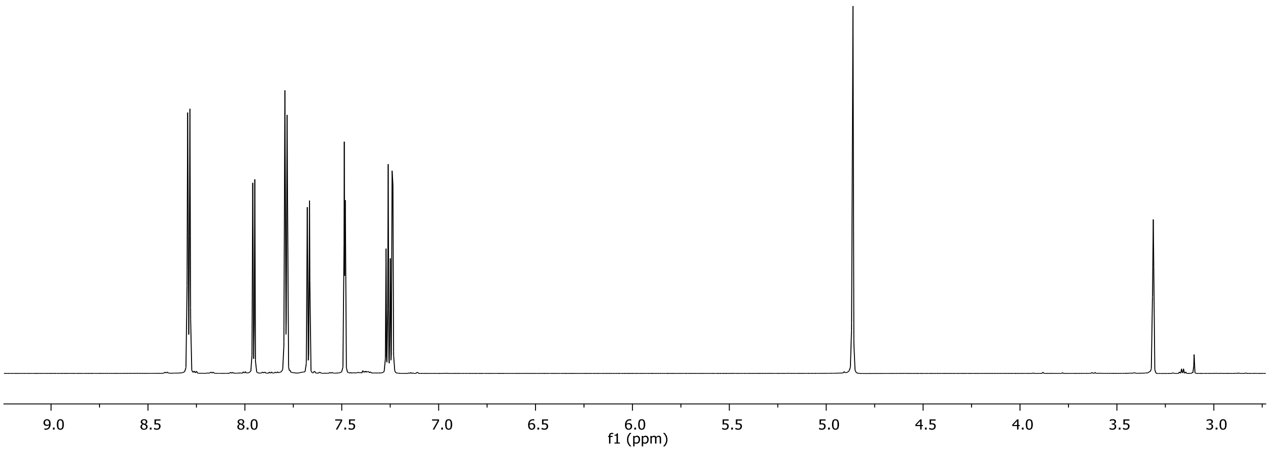

^{13}C NMR of compound **6** (175 MHz, CD_3OD)

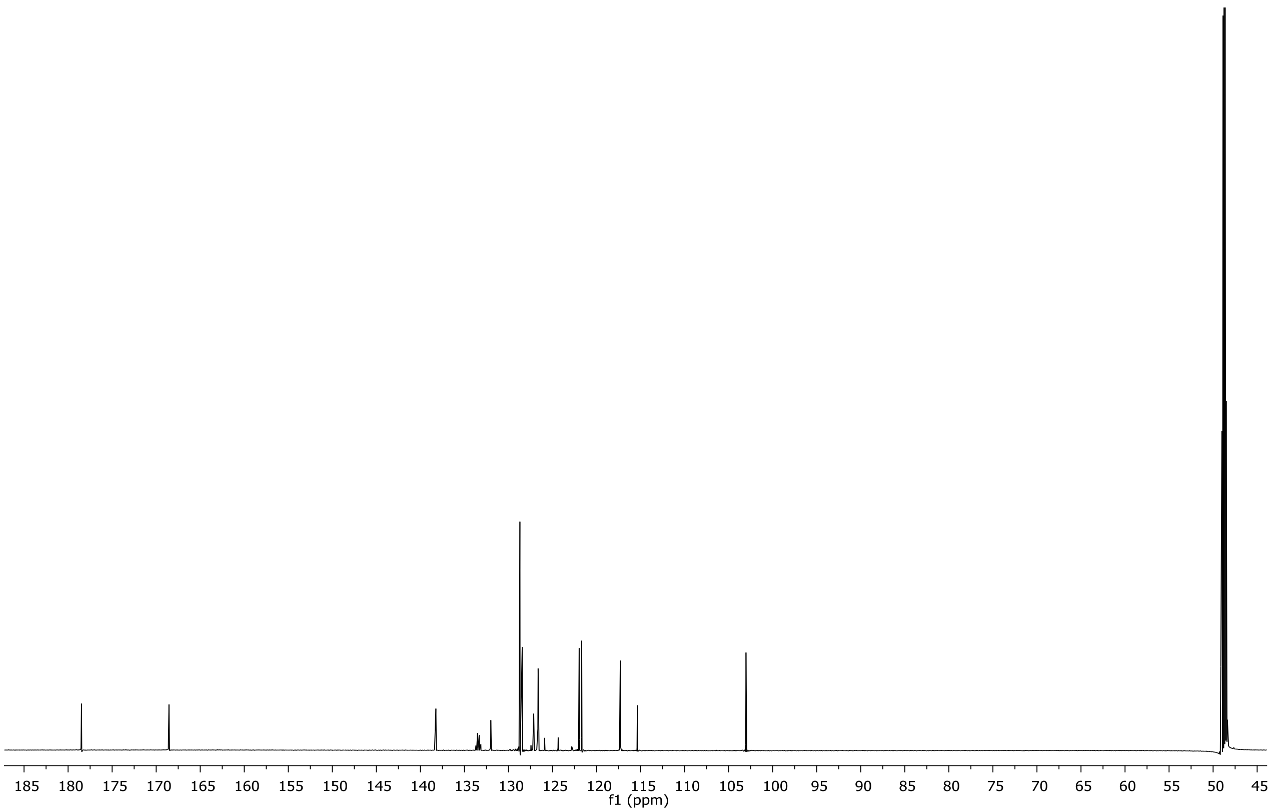

^{1}H NMR of compound **7** (400 MHz, CD_3OD)

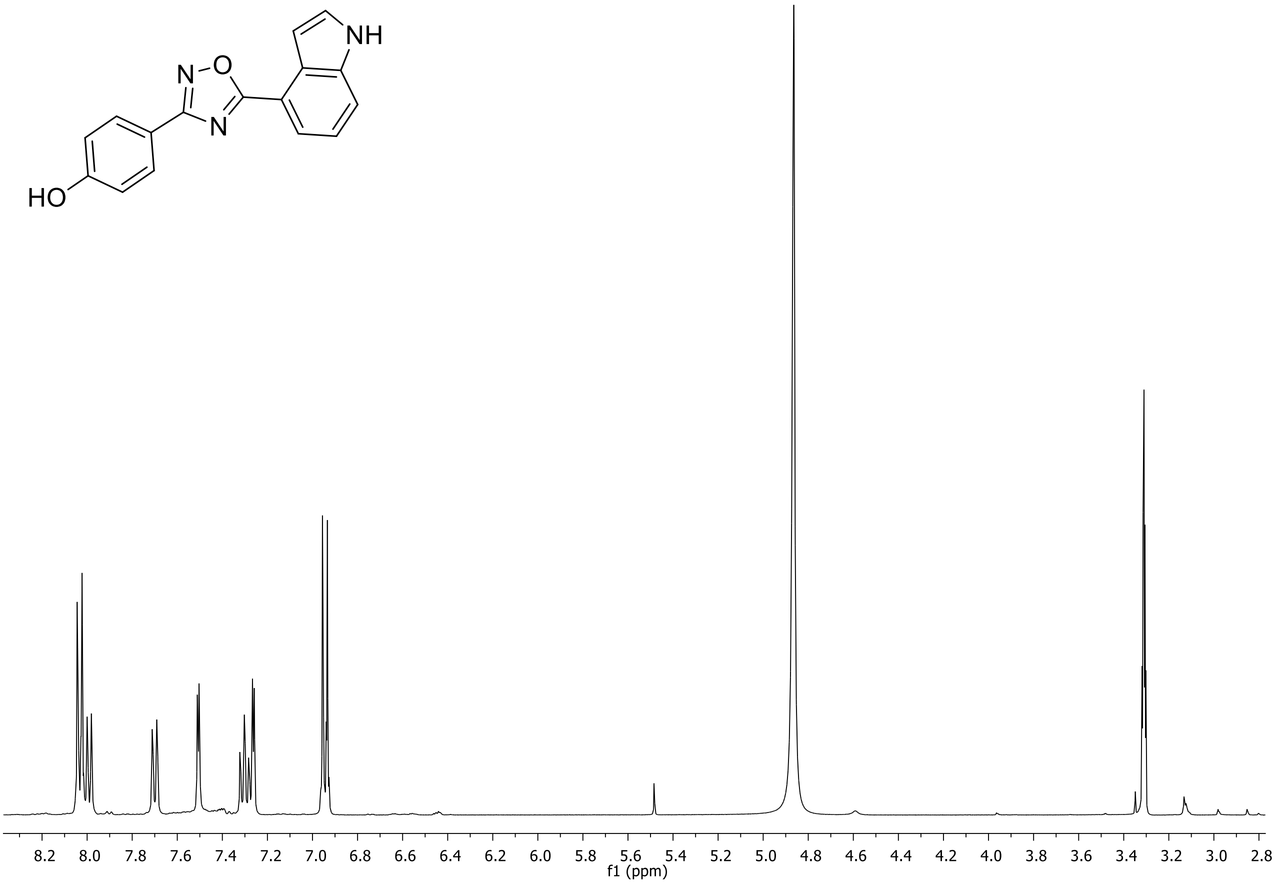

^{13}C NMR of compound **7** (100 MHz, CD_3OD)

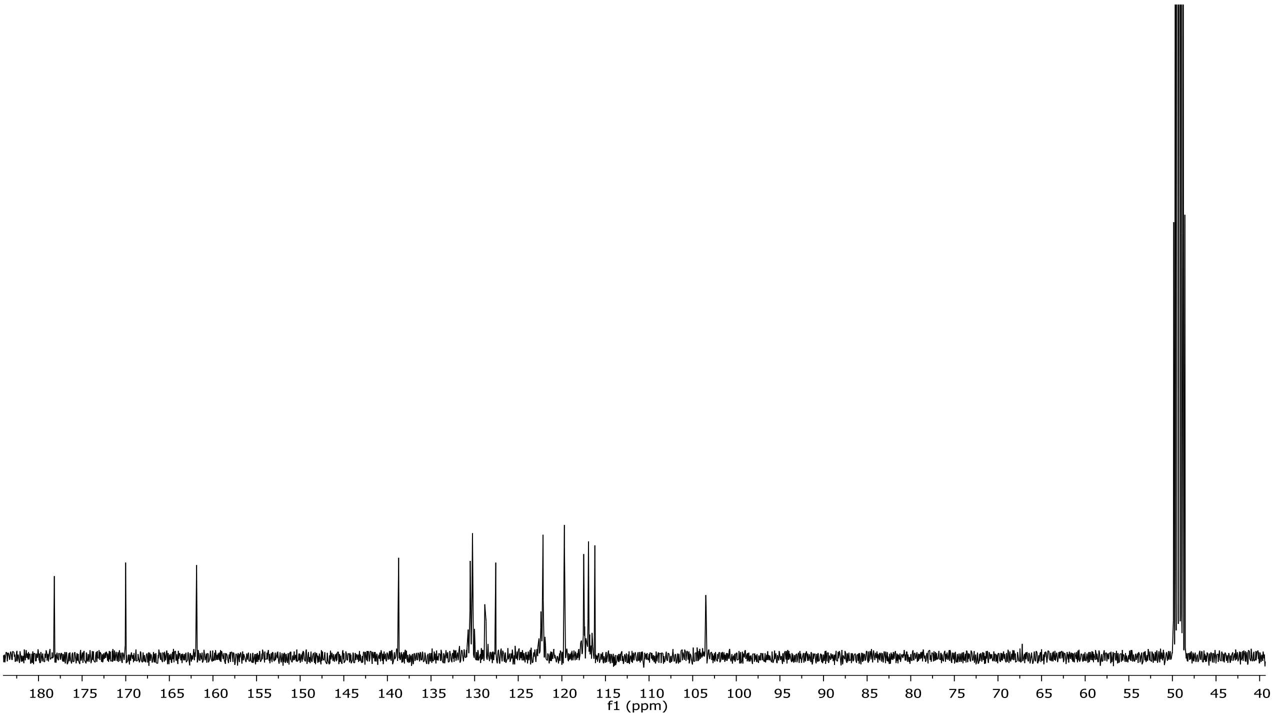

^{1}H NMR of compound **8** (700 MHz, CD_3OD)

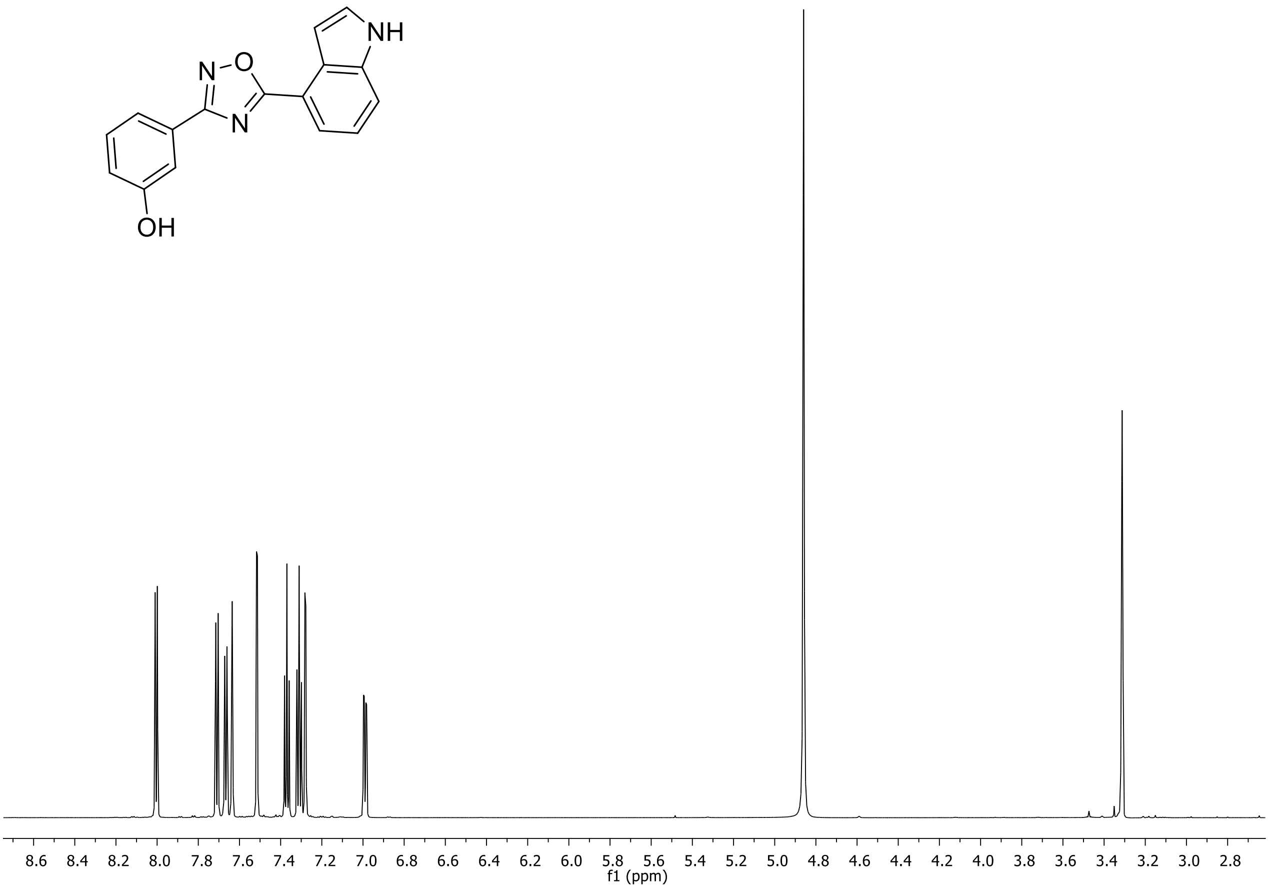

^{13}C NMR of compound **8** (175 MHz, CD_3OD)

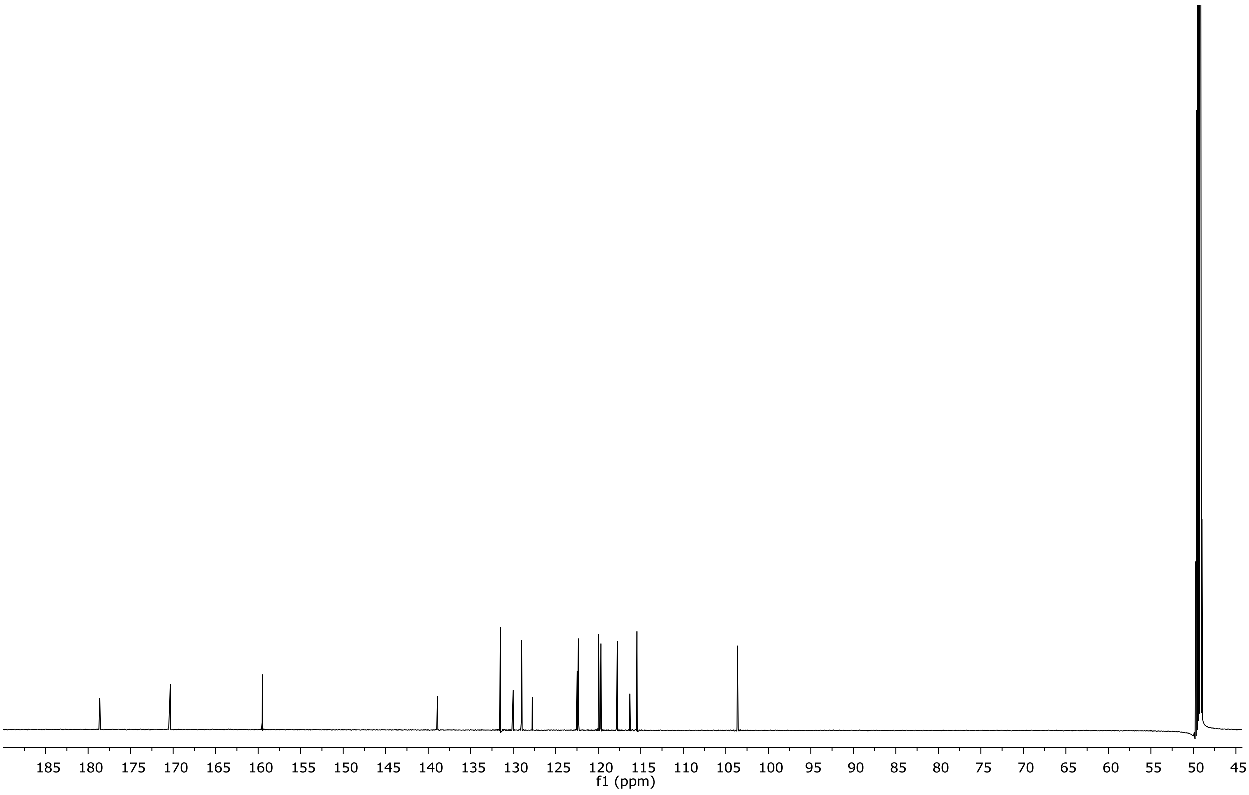

^{1}H NMR of compound **9** (400 MHz, CD_3OD)

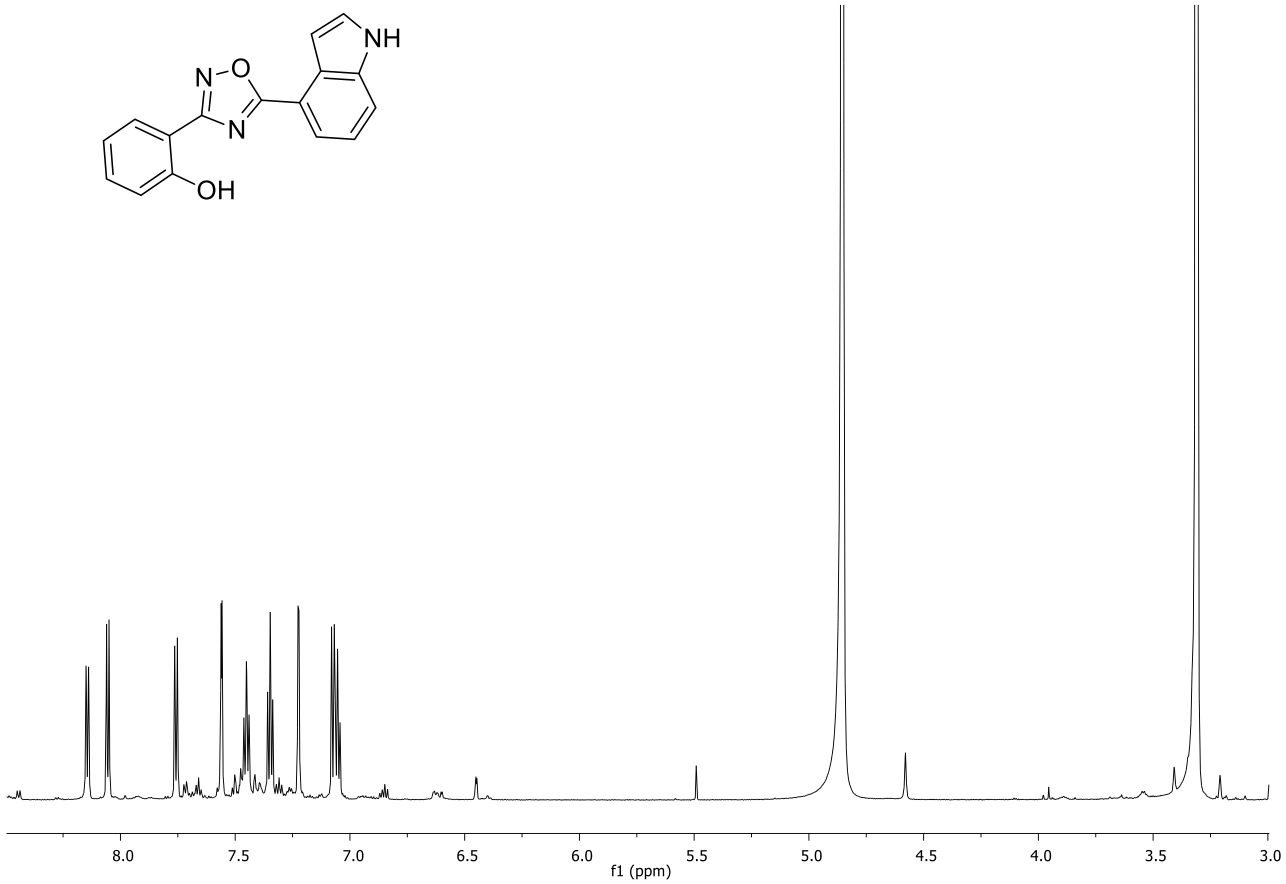

^{13}C NMR of compound **9** (100 MHz, CD_3OD)

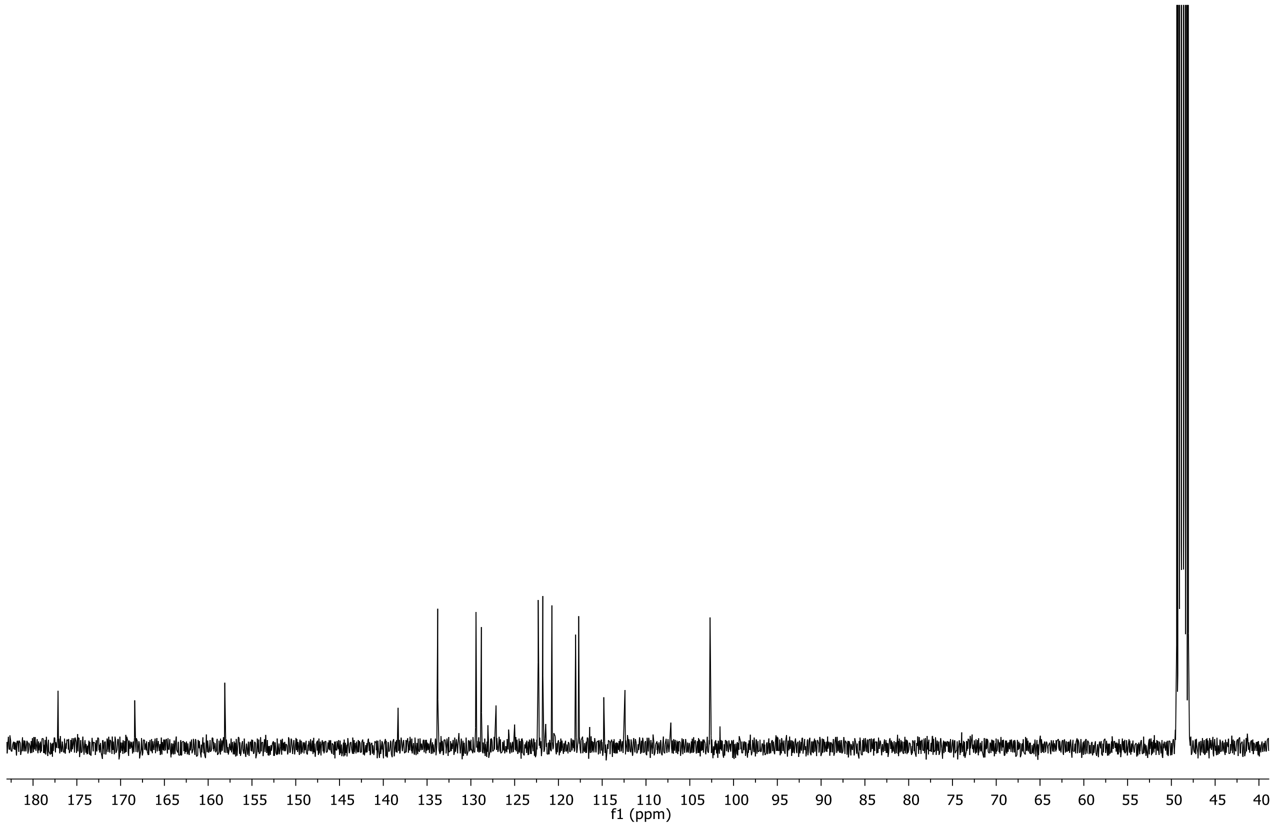

^{1}H NMR of compound **10** (400 MHz, CD_3OD)

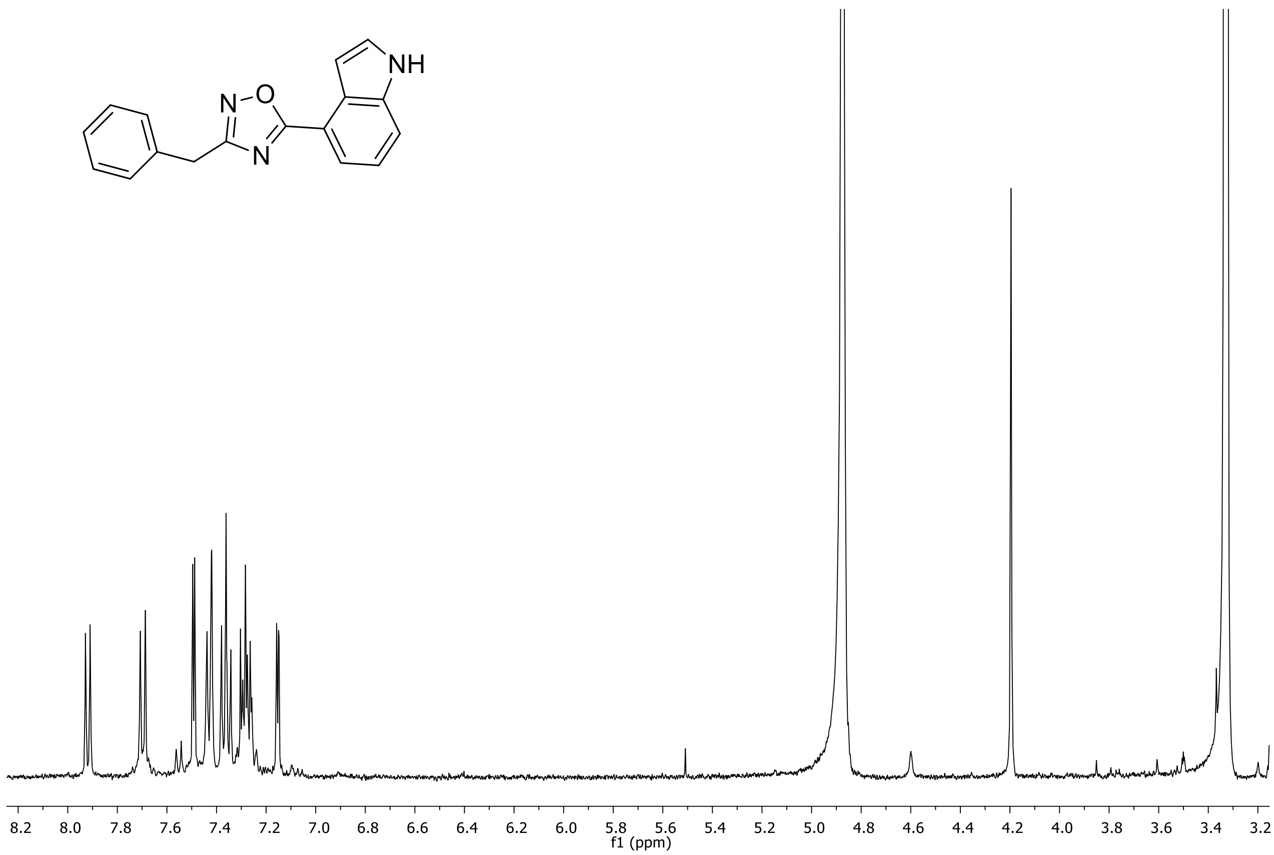

^{13}C NMR of compound **10** (175 MHz, CD_3OD)

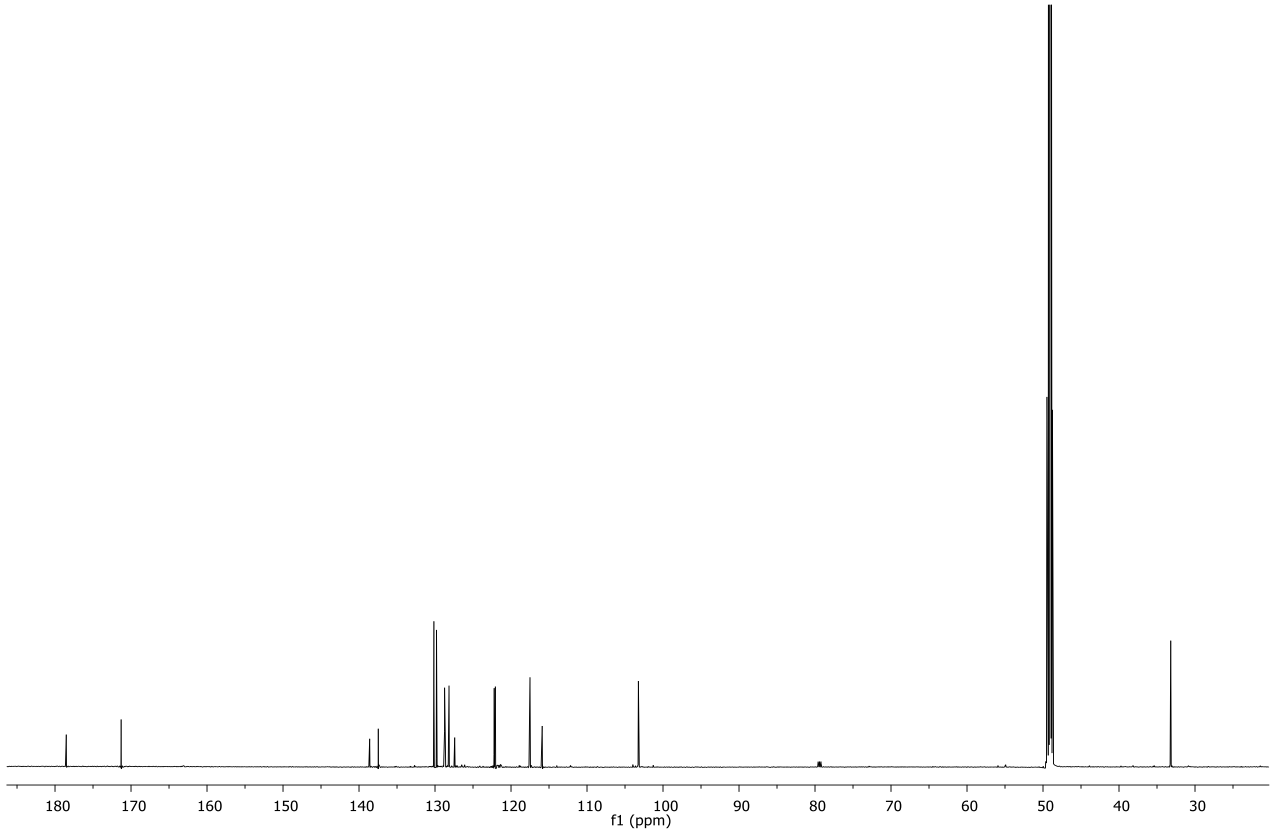

^{1}H NMR of compound **11** (700 MHz, CD_3OD)

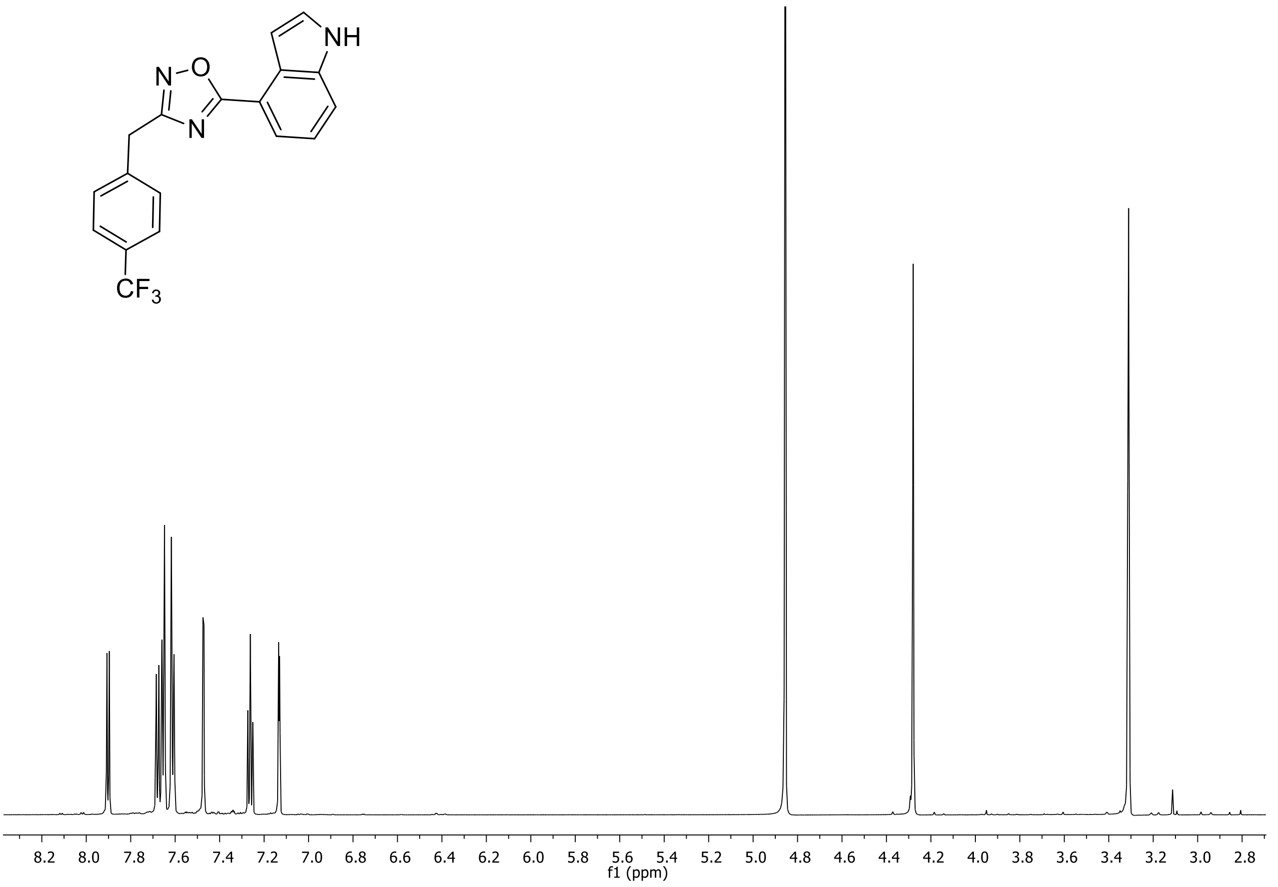

^{13}C NMR of compound **11** (175 MHz, CD_3OD)

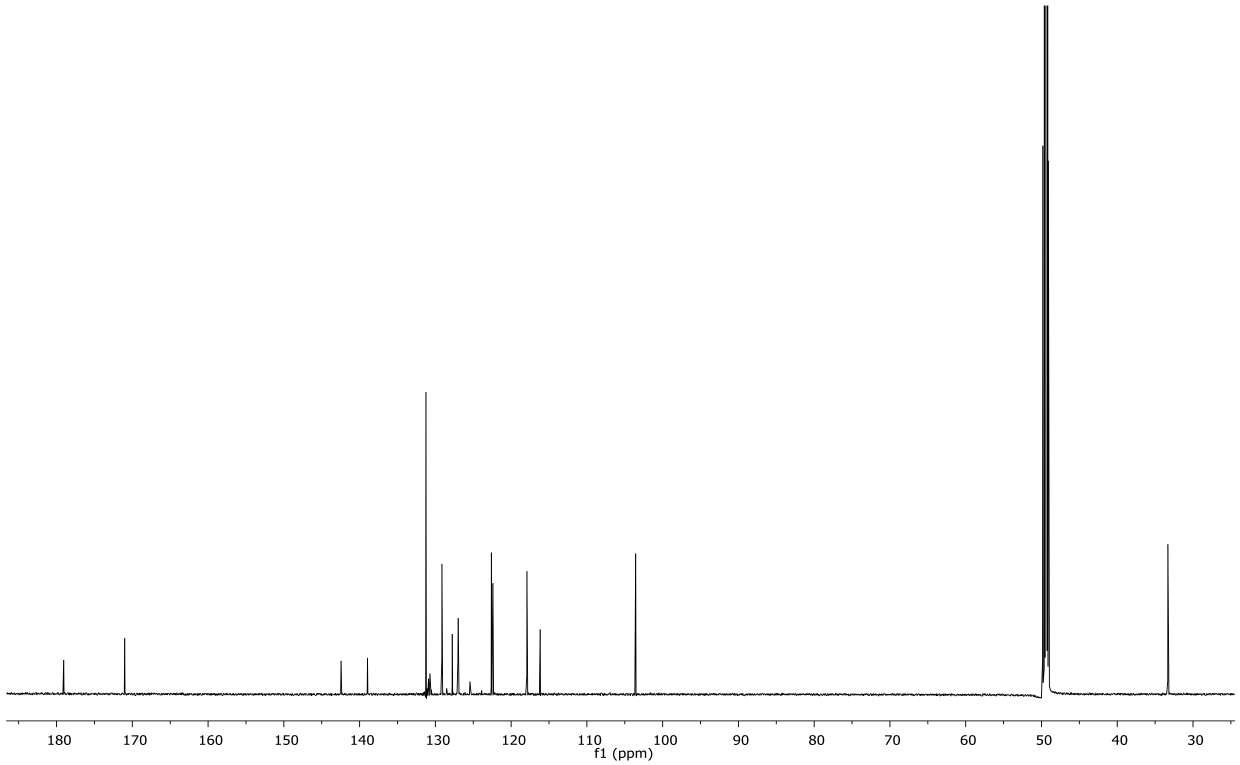

^{1}H NMR of compound **12** (700 MHz, CD_3OD)

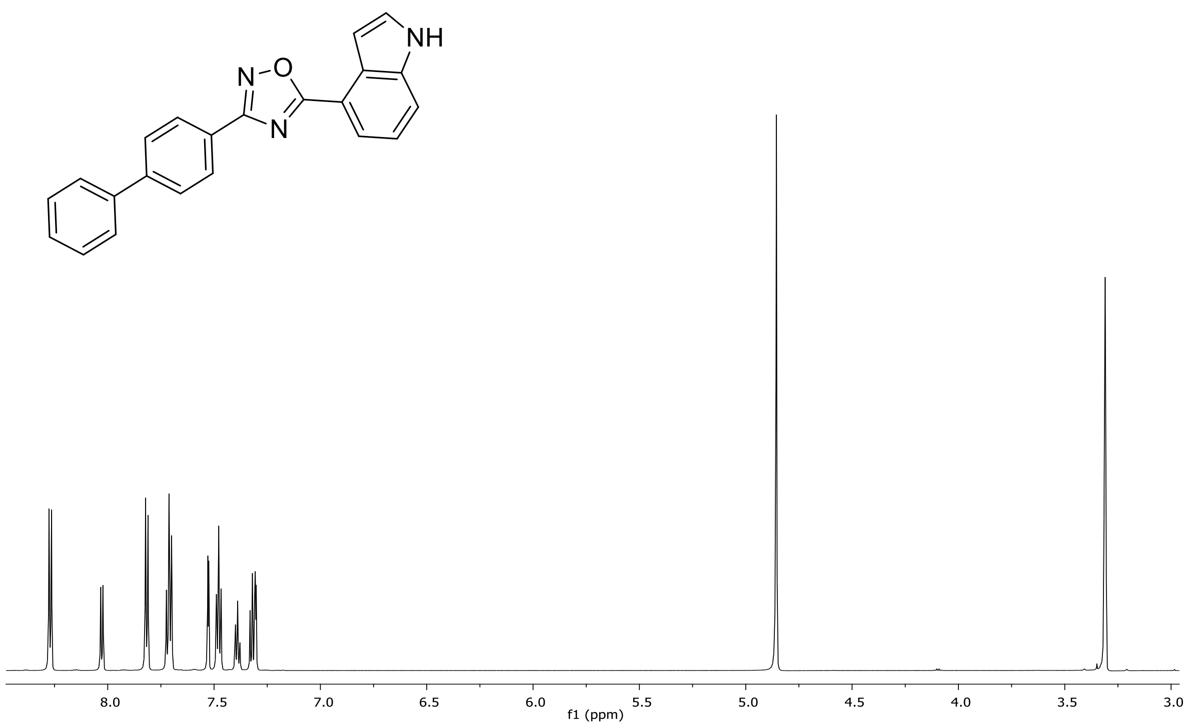

^{13}C NMR of compound **12** (175 MHz, CD_3OD)

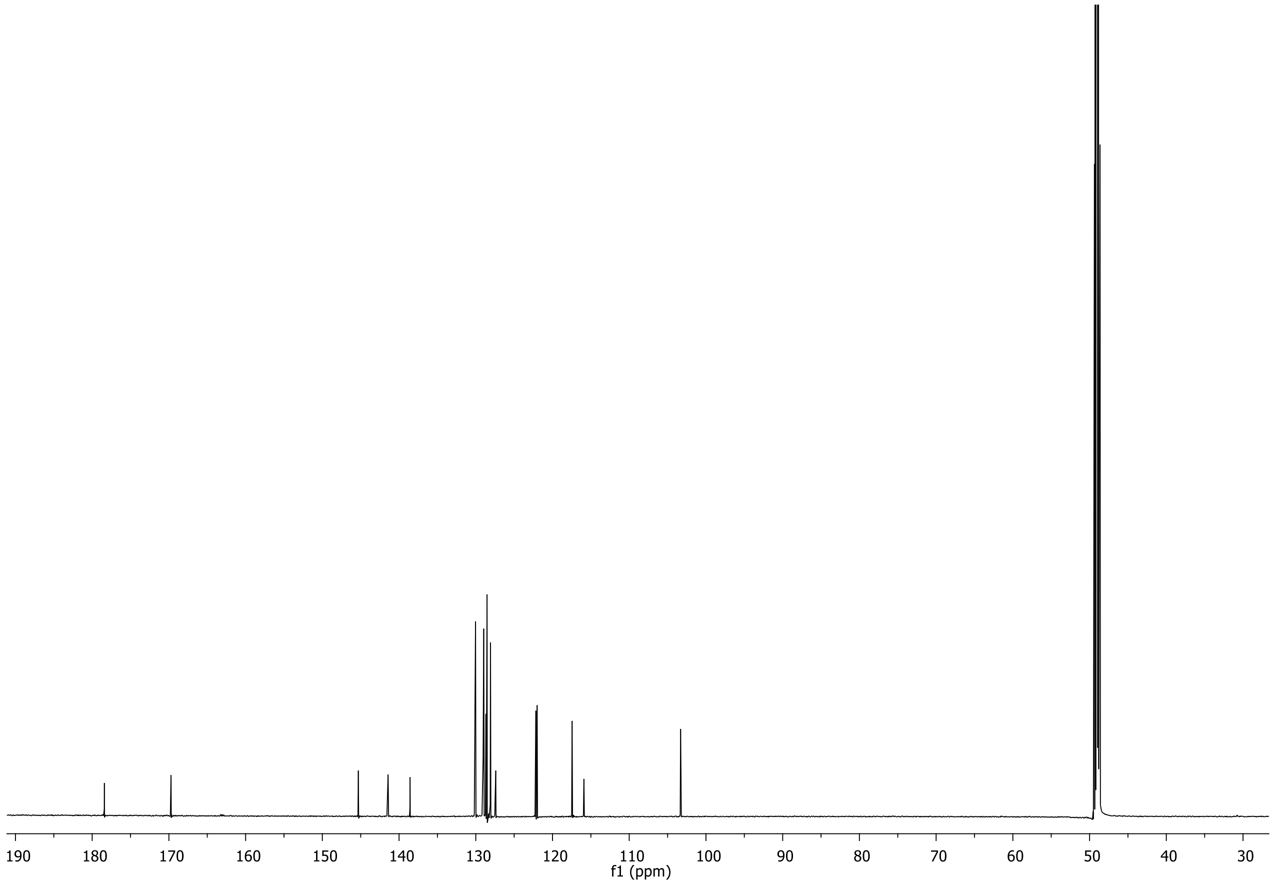

ESIMS spectrum of compound **12** (in positive mode)

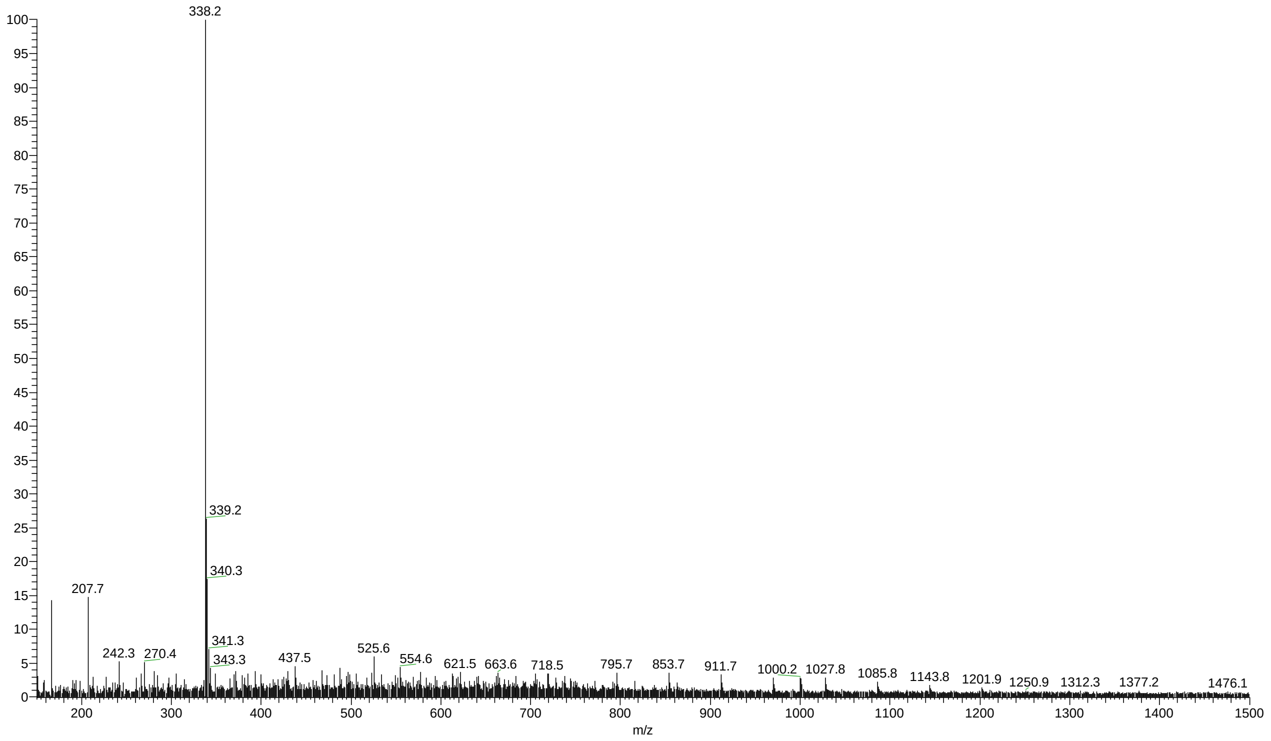

HPLC chromatogram of compound **12**

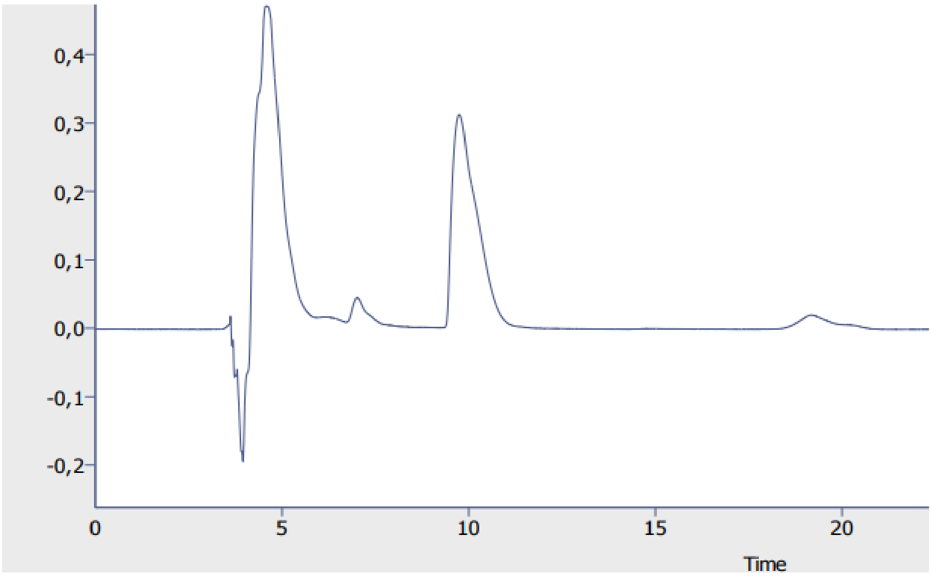

^{1}H NMR of compound **13** (400 MHz, CD_3OD)

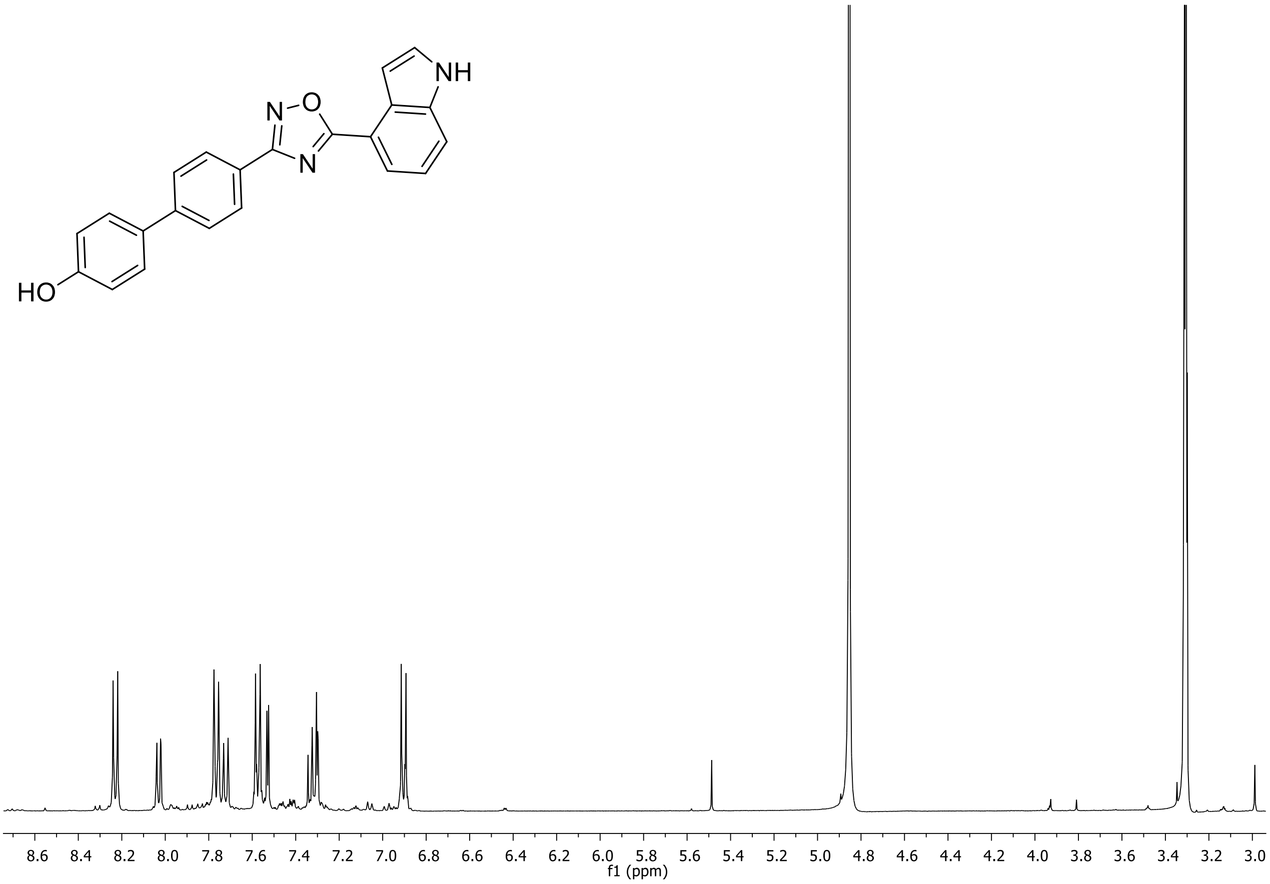

^{13}C NMR of compound **13** (100 MHz, CD_3OD)

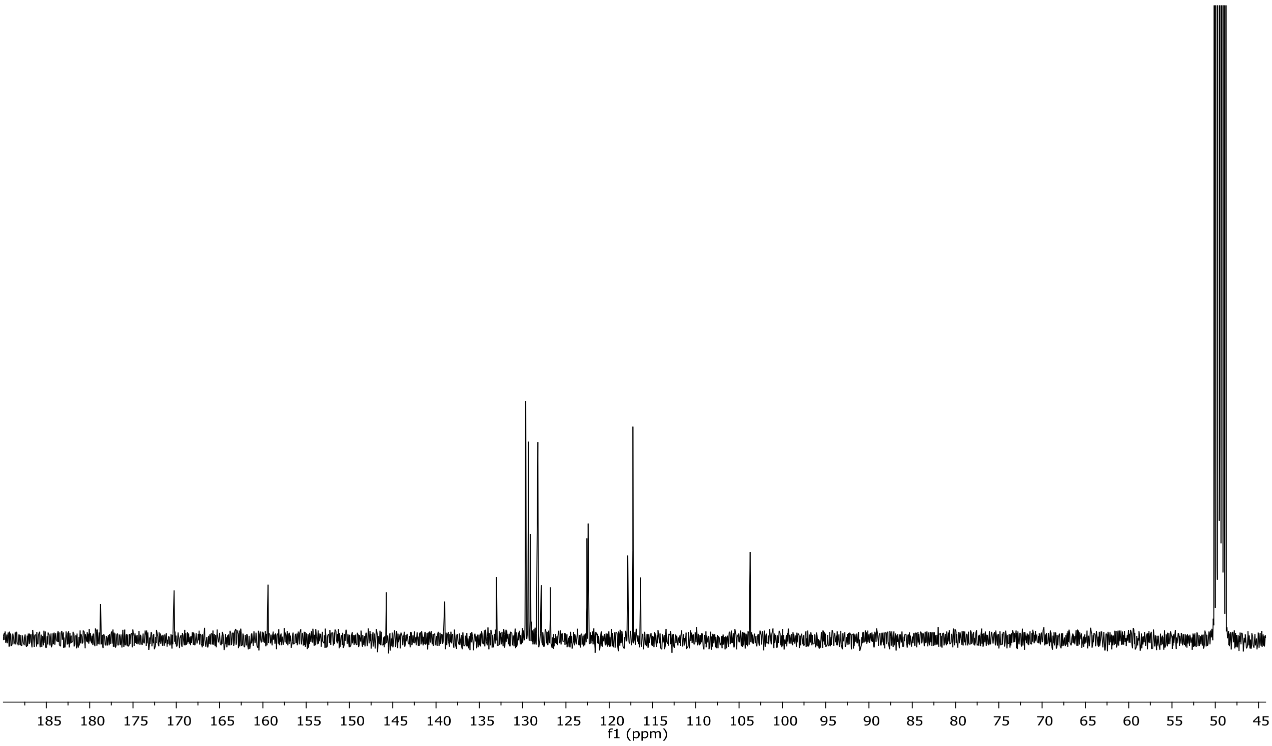

^{1}H NMR of compound **14** (700 MHz, CD_3OD)

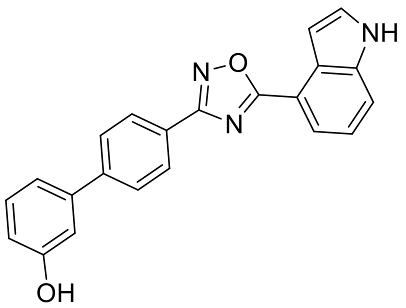

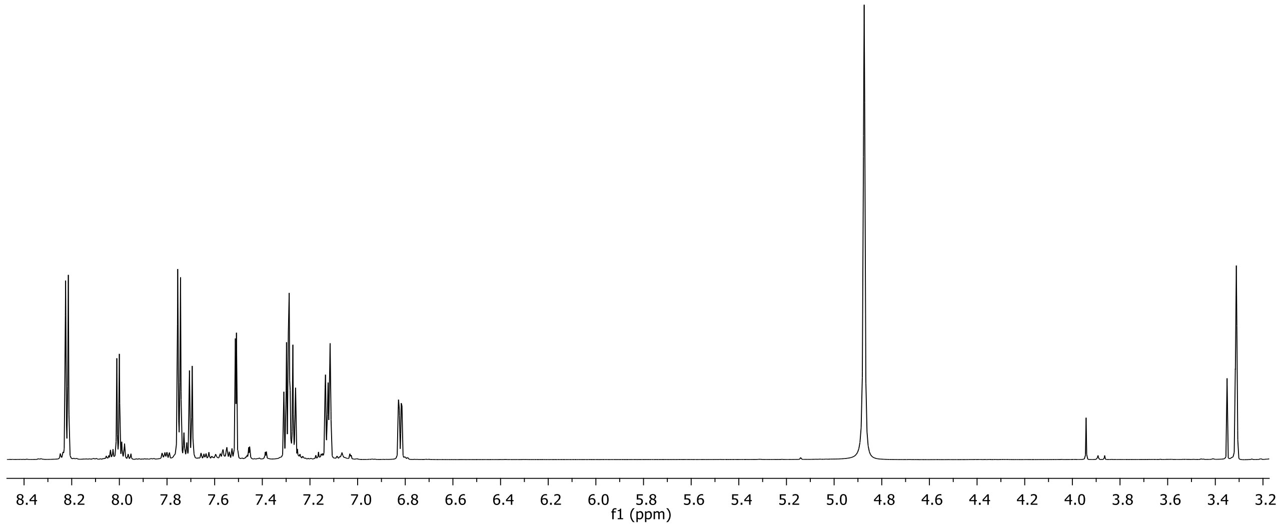

^{13}C NMR of compound **14** (175 MHz, CD_3OD)

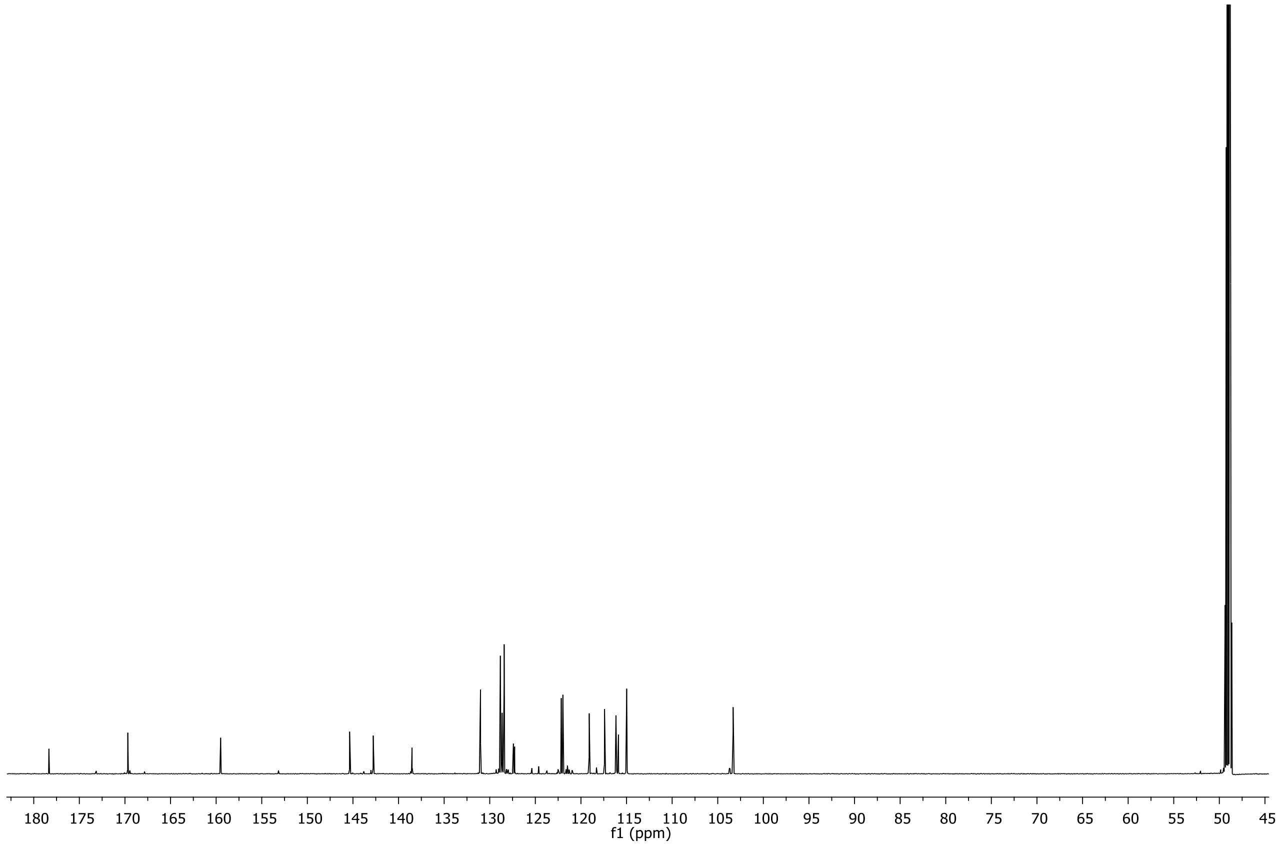

^{1}H NMR of compound **15** (700 MHz, CD_3OD)

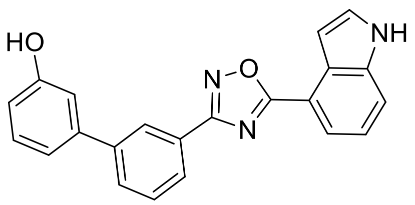

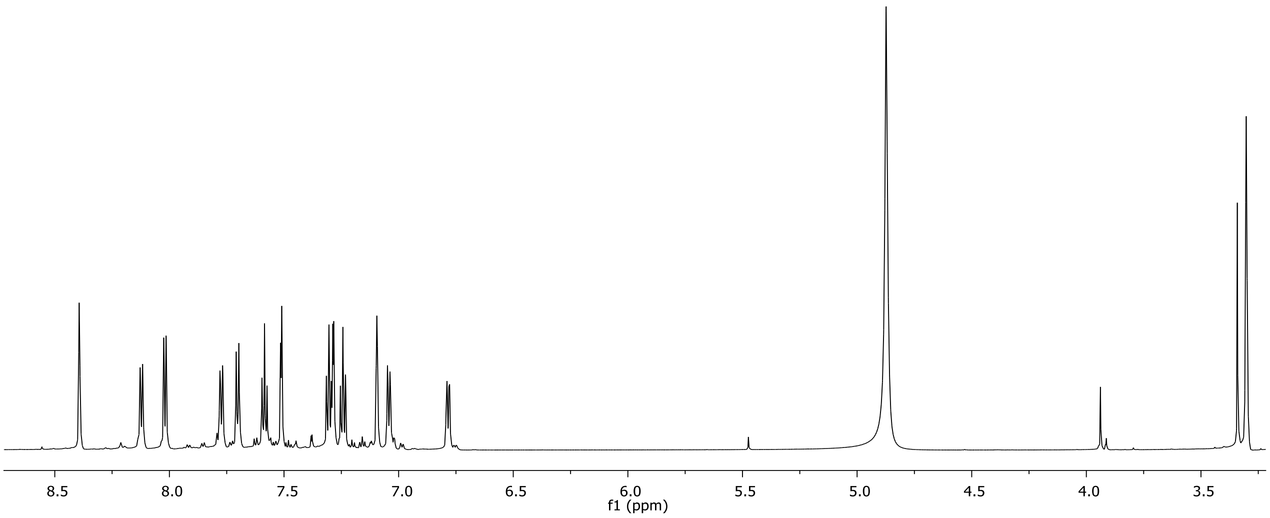

^{13}C NMR of compound **15** (175 MHz, CD_3OD)

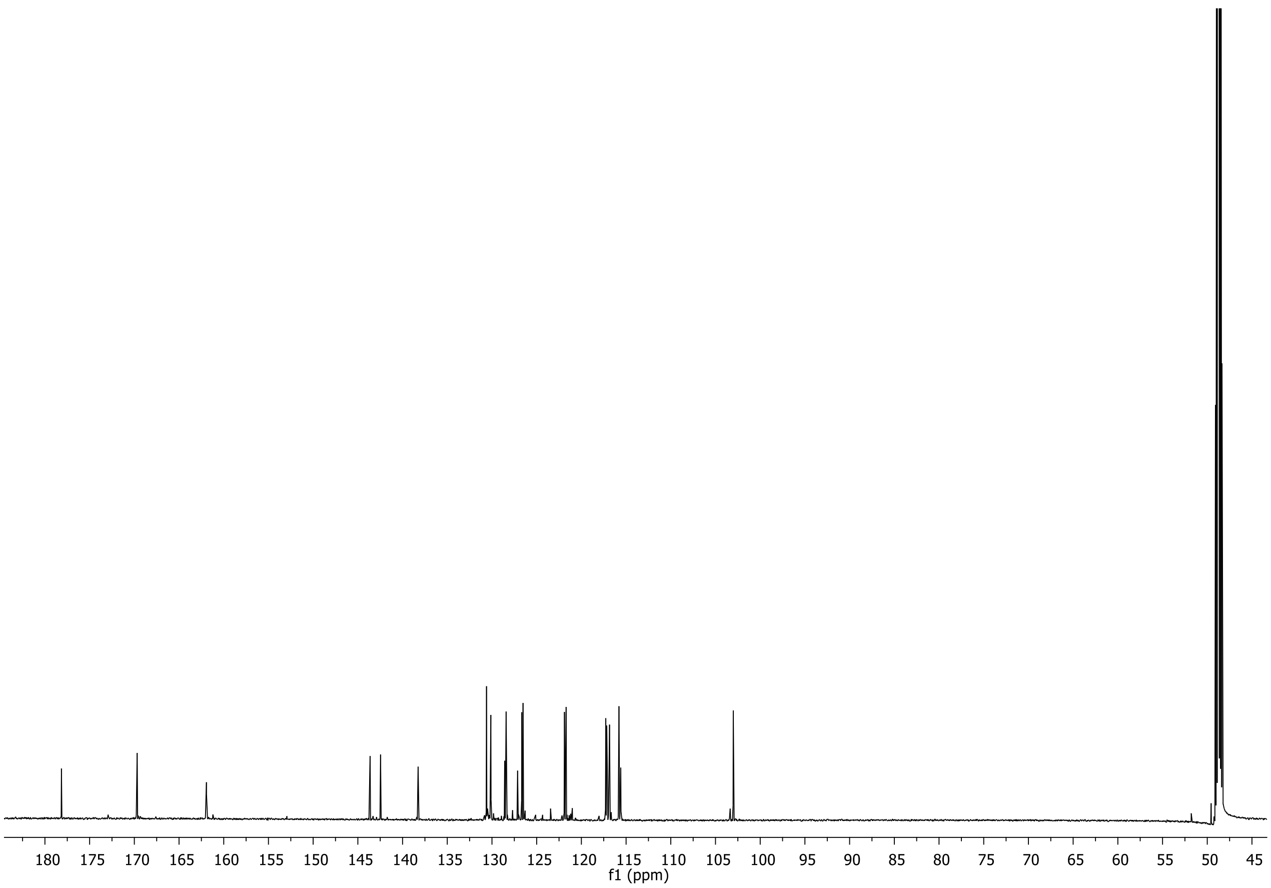

^{1}H NMR of compound **16** (400 MHz, $CDCl_3$)

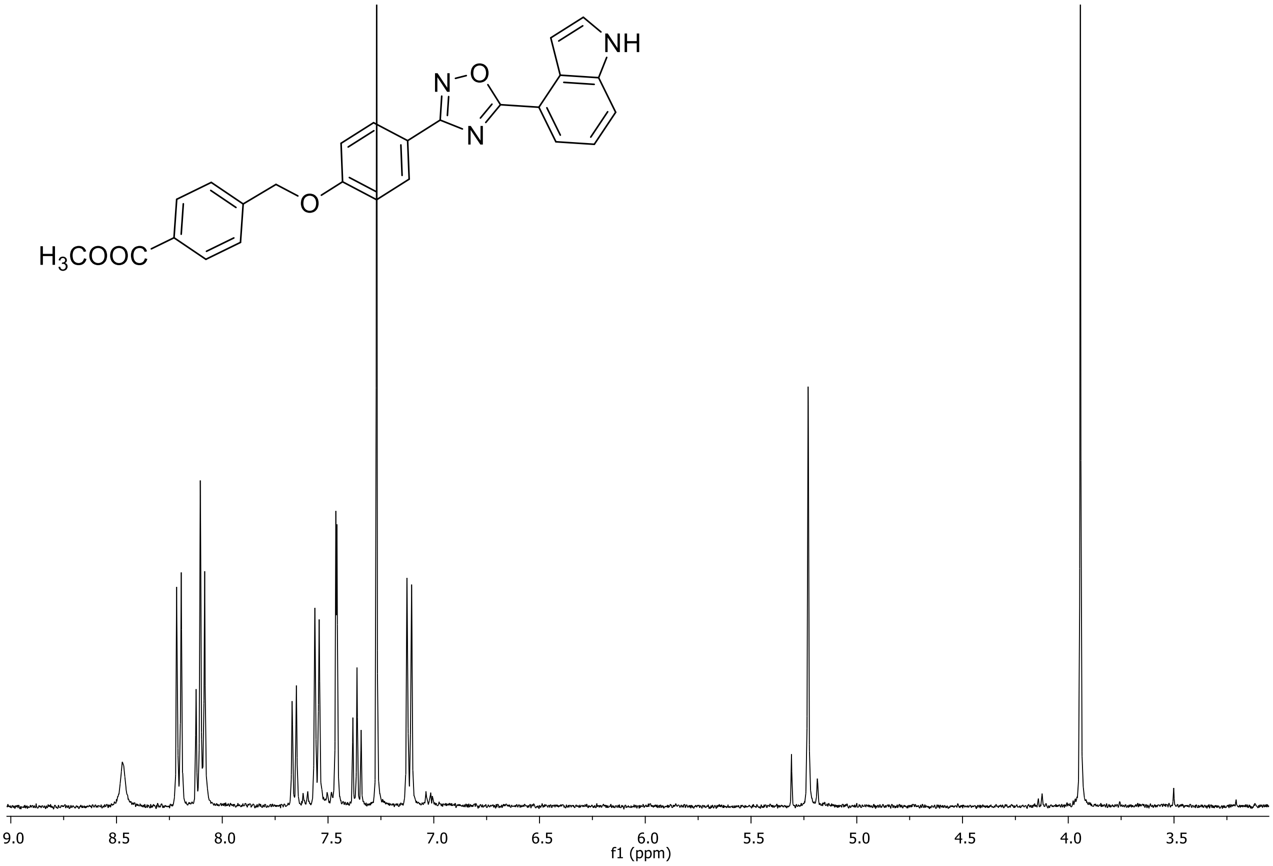

^{13}C NMR of compound **16** (100 MHz, $CDCl_3$)

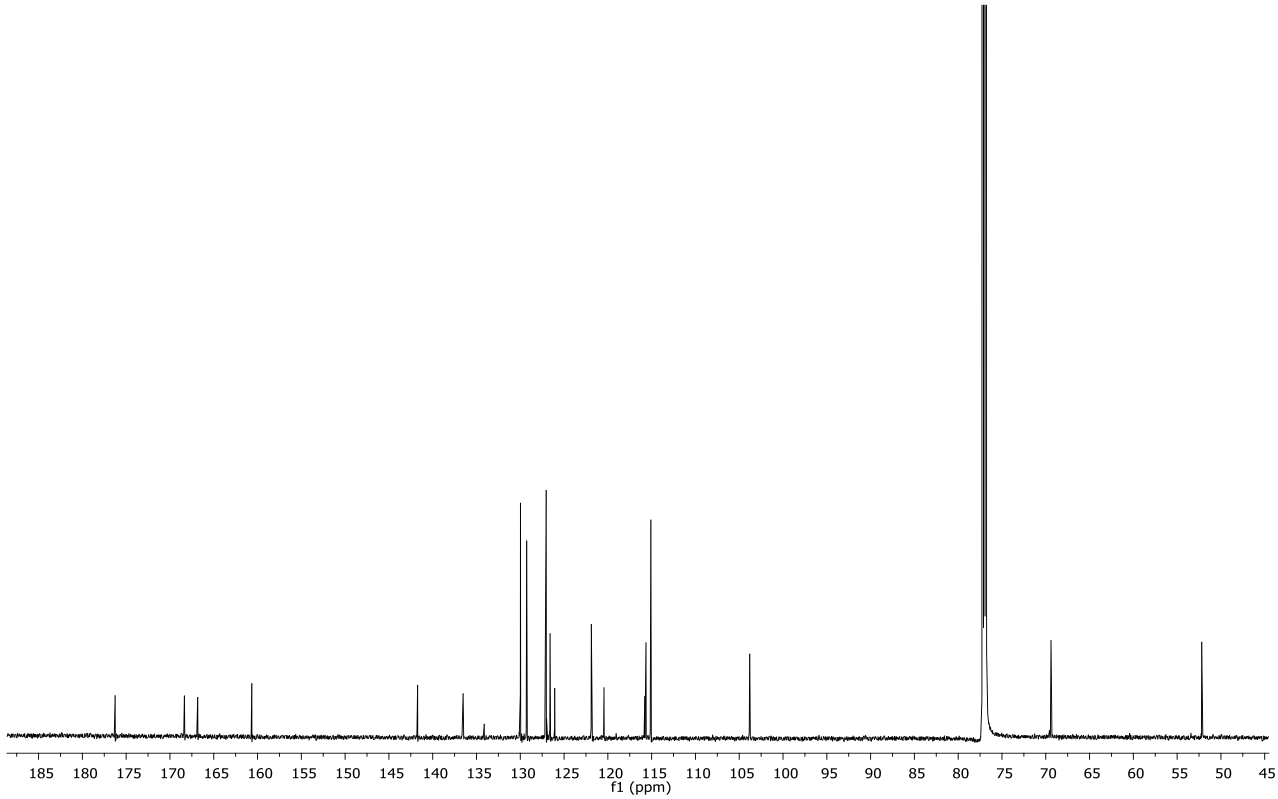

^{1}H NMR of compound **17** (400 MHz, $CDCl_3$)

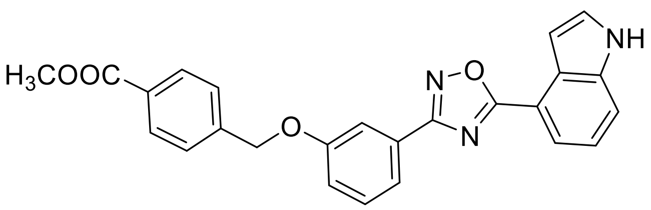

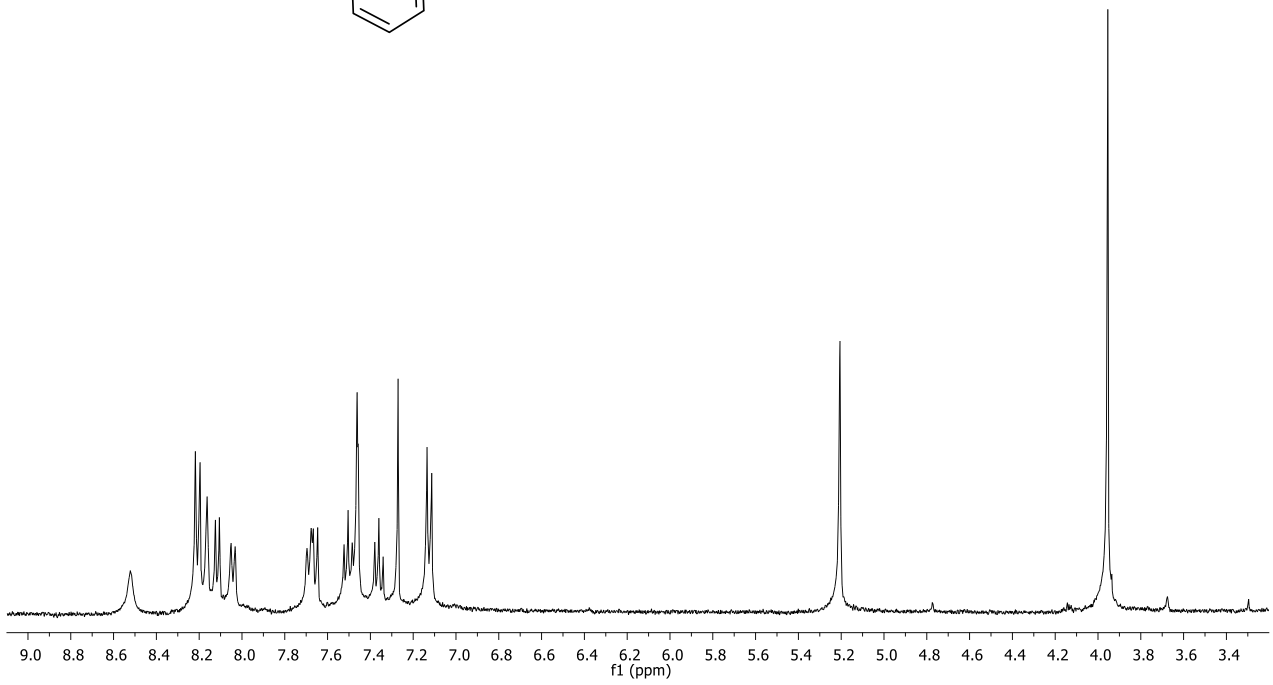

^{13}C NMR of compound **17** (100 MHz, $CDCl_3$)

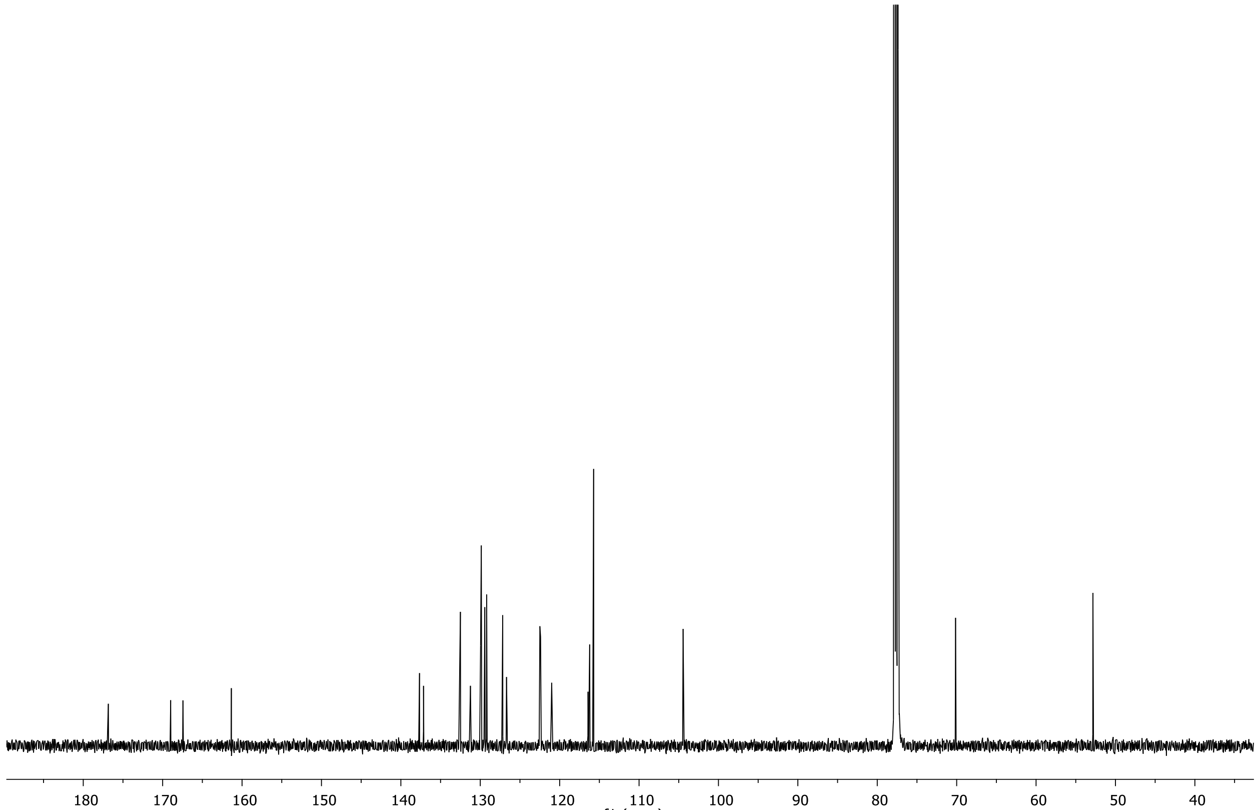

Supplement: Supplementary file 1 [file antibiotics-10-01258-s001.zip › antibiotics-1419636-supplementary.pdf]
